# The geroscience hypothesis: Economic paradigms and pharmacological strategies for healthspan extension

**DOI:** 10.1016/j.jtumed.2026.02.004

**Published:** 2026-02-25

**Authors:** Adel F. Alotaibi, Areej I. Aljasser, Abdullah N. Alkattan, Ahmed Alkhoraisi, Abdullah M. Assiri

**Affiliations:** Deputyship of Population Health, Ministry of Health, Riyadh, KSA

Dear Editor,

The socioeconomic implications of an aging global population have catalyzed a shift in the biomedical sciences toward “geroscience.” This paradigm posits that since aging is the primary risk factor for most chronic diseases, targeting the molecular hallmarks of aging will delay the onset of multiple morbidities simultaneously. Despite the theoretical robustness of this approach, translating preclinical findings into human therapeutics requires navigating a complex landscape of funding, trial design, and regulatory hurdles.^1^

## Economic paradigms in geroscience

The financing of longevity research has evolved from fragmented academic grants into a sophisticated economic sector defined by three distinct funding models. First, outcome-driven and prize-based incentivization model. The XPRIZE Healthspan and the National Academy of Medicine (NAM) Healthy Longevity Global Grand Challenge are two examples of initiatives that follow this type of model, which addresses the high failure rates of early-stage research. By offering over $100 million in prize funding, these initiatives incentivize the achievement of specific functional benchmarks, such as muscle and immune restoration, thereby drawing interdisciplinary expertise into the field. This “push–pull” mechanism, where “push” funding promotes initial research costs and “pull” incentives offer rewards for successful outcomes, provides non-dilutive financing that de-risks high-stakes, high-reward inquiries.^1^^,^^2^ In contrast, “moonshot” entities like Altos Labs and Calico Life Sciences represent the large-scale corporate venture and vertical integration model. These large biotechnology companies utilize massive capital investments (e.g., $3 billion for Altos) to internalize the basic science, concentrating on crucial areas like cellular reprogramming, which focuses on epigenetic rejuvenation to reset cellular age without loss of somatic identity, and strategic alliances.^1^^,^^3^^,^^4^ An example of the latter is the Calico-AbbVie collaboration, which exemplifies a hybrid model where geroscience discovery is paired with established pharmaceutical infrastructure for clinical development and commercialization. Furthermore, the Hevolution Foundation represents a novel model of catalytic philanthropy. With an annual commitment of $1 billion, it serves as a global catalyst specifically designed to bridge the “valley of death,” the funding gap between preclinical research and clinical trials. This is achieved by supporting early-stage discovery through the Hevolution Foundation Geroscience Research Opportunities (HF-GRO) international initiative and, at the same time, financing studies conducted among human subjects. These studies are essential for converting high-potential preclinical laboratory findings into viable clinical pipelines. Such an approach ensures a robust scientific route that remains resilient against the market volatility or shifting priorities of individual corporate entities.^2^

## Pharmacological strategies: targeting the hallmarks of aging

Therapeutic interventions are increasingly categorized by their mechanism of action within the aging process, moving beyond simple primary prevention toward biological age deceleration.^3^, ^4^, ^5^, ^6^ One main area of focus is senotherapeutics, which includes both senolytics and senomorphics. The accumulation of senescent cells, characterized by the Senescence-Associated Secretory Phenotype (SASP), drives a chronic state of “inflammaging”, which is defined as the age-related increase in systemic, sterile pro-inflammatory markers that acts as a primary contributor for multiple morbidities. Senolytics are agents that induce apoptosis in senescent cells by inhibiting Senescent Cell Anti-Apoptotic Pathways (SCAPs). The dasatinib and quercetin (D + Q) combination remains the most studied, with trials investigating efficacy in idiopathic pulmonary fibrosis and chronic kidney disease. Yet, senomorphics are agents, such as metformin and rapamycin, that modulate the SASP without inducing cell death. This strategy offers a potentially higher safety profile for long-term prophylactic use. In addition, targeting evolutionarily conserved nutrient-sensing and metabolic pathways that regulate energy homeostasis is a primary pillar of geroscience. This is evidenced by two mechanisms: inhibition of the mechanistic target of rapamycin (mTOR) and activation of AMP-activated protein kinase (AMPK). Rapamycin and its analogs (rapalogs) are the gold standard for healthspan extension in model organisms. Current research focuses on selective mTOR complex 1 (mTORC1) inhibition to minimize metabolic side effects like insulin resistance. Besides, activating the AMPK pathway via metformin has led to the Targeting Aging with Metformin (TAME) trial, a pivotal study designed to establish a regulatory precedent for aging as a targetable clinical indication. Additionally, strategies targeting genomic stability and mitochondrial support focus on nicotinamide adenine dinucleotide (NAD^+^) augmentation. As cellular NAD^+^ levels decline with age, the activity of NAD^+^-dependent enzymes, mainly sirtuins and poly(ADP-ribose) polymerases, is impaired, compromising DNA repair. Clinical interest in NAD^+^ augmentation, like NAD^+^ precursors (nicotinamide mononucleotide (NMN) and nicotinamide riboside (NR)), aims to restore mitochondrial energetics and maintain genomic integrity.

## Challenges and future directions

The transition of healthy longevity into a major economic sector is hampered by the lack of validated surrogate endpoints and the traditional “one drug, one disease” regulatory framework.^1^^,^^3^^,^^6^ To achieve the 38 trillion US dollar societal benefit projected to result from a one-year extension of healthy life expectancy,^7^ several systemic shifts are required. First, there should be a standardization of biomarkers to establish validated measures of biological age (clocks) based on epigenetic, proteomic, or functional markers that are accepted by regulatory bodies, such as the United States Food and Drug Administration (USFDA) and the European Medicines Agency (EMA). Accompanying this is the need for trial design innovation, moving toward multi-morbidity endpoints rather than single-disease outcomes to capture the systemic benefits of geroscience. Finally, global policy alignment is vital to leverage international foundations and harmonize the pathway from discovery to clinical application, ensuring equitable access to longevity interventions.

## Ethical approval

Not applicable.

## Authors contributions

AO, AKH, and AA contributed in conceptualization. AJ and AKA contributed in writing-original draft preparation. AKH and AA contributed in writing-review and editing. AO and AJ, and AKA contributed in resources. All authors have critically reviewed and approved the final draft and are responsible for the content and similarly index of the manuscript.

## Source of funding

This research did not receive any specific grant from funding agencies in the public, commercial, or not-for-profit sectors.

## Conflict of interest

The authors declare no conflict of interest.
